# Arthroscopic Biportal Visualization and “Precise Resection” for Recalcitrant Lateral Epicondylitis Lesion

**DOI:** 10.1016/j.eats.2025.103862

**Published:** 2025-09-12

**Authors:** Chuan Zhang, Hai-Feng Gu, Jiang-Tao Ma, Ming-Ming Dong, Sui-Zhu Huang

**Affiliations:** a Luoyang Orthopedic Hospital of Henan Province and Orthopedic Hospital of Henan Province, Henan University of Chinese Medicine, Zhengzhou, China; b Zhejiang Provincial People’s Hospital, Hangzhou, China

## Abstract

Refractory lateral epicondylitis poses a significant clinical challenge. In patients with persistent symptoms refractory to conservative treatment, surgical intervention represents an effective approach for achieving durable symptomatic relief. Advances in arthroscopic techniques now offer a more minimally invasive and refined approach. While there is no consensus regarding the superiority of one arthroscopic treatment over another, resection of the lesion tissue remains a critical consideration. This article describes an arthroscopic extensor carpi radialis brevis tendon resection technique, which facilitates precise visualization and targeted resection of pathological tendon tissue, avoiding excessive resection of healthy tissue or incomplete resection of pathological lesions, while simultaneously addressing any concomitant intra-articular pathology.

Surgical resection of the extensor carpi radialis brevis (ECRB) tendon remains the standard treatment for refractory lateral epicondylitis. Contemporary surgical management strategies for this condition include both open and arthroscopic release of the ECRB tendon, with each approach showing clinical efficacy in pain relief and functional restoration.^1^ Notably, the arthroscopic technique offers superior intra-articular visualization and typically facilitates accelerated postoperative rehabilitation.^2^ However, persistent challenges in achieving precise ECRB resection under arthroscopy necessitate further technical refinement. To address this critical limitation, we present an arthroscopic biportal technique that integrates enhanced visual evaluation with precise lesion tissue resection. We assert that utilizing the anterolateral portal to reassess lesion tissue located anterior to the equatorial line of the capitellum provides several advantages: it allows for accurate location of the equatorial line and precise excision of pathological tissue, reduces the likelihood of residual postoperative pain, and promotes a fast recovery time.

## Surgical Technique

The surgical technique is shown in Video 1.

### Indications and Preoperative Planning

The procedure is indicated for the following situations: lateral epicondylitis that is refractory to conservative treatment and has persisted for more than 6 to 8 months, as well as severe disabling pain that interferes with sleep and is not alleviated by conservative methods, represent relative indications. Patients exhibiting tenderness and lesion distribution restricted anterior to the middle line radial head are also candidates for this procedure.

In cases where patients have previously undergone anterior transposition of the ulnar nerve or have a history of medial elbow open injury or prior surgery, this technique may be considered relatively indicated. Alternatively, surgical exposure of the ulnar nerve through an open incision can be performed before establishing the medial arthroscopic portal. Additionally, cases involving confirmed ulnar nerve anterior subluxation or snapping medial triceps identified via preoperative ultrasound may also benefit from medial open ulnar nerve exposure prior to establishing the medial portal.

Conversely, patients whose lesions and tenderness are extensive posteriorly to the extensor digitorum communis and posteriorly over the radial head middle line are not suitable candidates for this technique. Furthermore, individuals who have inadequately responded to nonoperative treatments or shown a lack of compliance with activity modification should also be excluded from consideration.

### Patient Positioning and Setup

**Fig 1:**
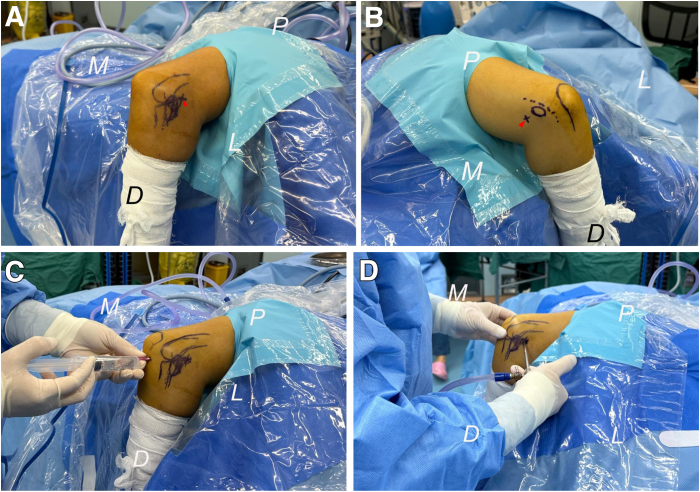
Photographs showing views of the right elbow in a patient positioned laterally. (A) Prior to anesthesia, the radial head and capitellum are identified, and the tender area (black oval circle) anterior to the equatorial line (black dotted line) of the radial head is marked. Additionally, the planned anterolateral portal (white arrowhead) is also delineated. (B) Following exsanguination of the upper limb and inflation of the tourniquet, markings are made for the olecranon, medial epicondyle, ulnar nerve, and planned anteromedial portal (red arrowhead). (C) Approximately 20 mL of saline solution is injected into the joint cavity through the posterolateral “soft spot” to facilitate expansion of the elbow joint. (D) The anterolateral portal is created first with the trocar and sheath being inserted. (D, distal; L, lateral; M, medial; P, proximal.)

The patient is administered general anesthesia and placed in the lateral decubitus position, with the elbow joint supported by an arm holder as proximally as possible. A nonsterile tourniquet is applied in a similar manner and inflated immediately after sterile draping. Prior to anesthesia administration, while the patient remains conscious, the tender area that precisely indicates the location of pathological changes is outlined. The other anatomic landmarks of the elbow and planned portals can be delineated after sterile draping (Fig 1).

### Portal Creation

The planned anterolateral portal is situated directly anterior to the radiocapitellar joint, commonly referred to as the mid-anterolateral portal. This location is easily palpable at the anterolateral “soft spot,” which is formed by the distal lateral humeral column and radial head when the elbow is flexed at 90°.

**Fig 2:**
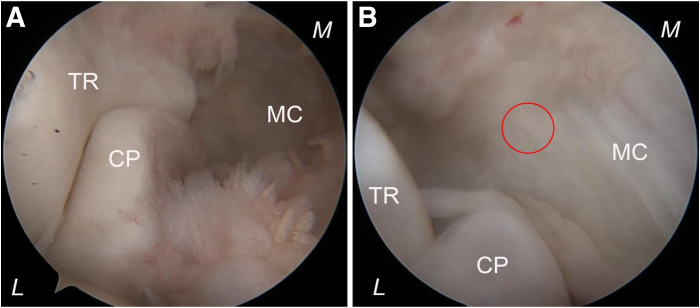
Arthroscopic photographs demonstrating views from the anterolateral portal for the right elbow case in a lateral decubitus position. (A) The anterolateral portal is created first. The trochlea (TR) and coronoid process (CP) are inspected for a potential lesion while viewing from the lateral side. (B) The arthroscope is pushed alongside the trochlea toward the medial elbow capsule, indicated by the red circle, and further advanced through the capsule and the flexor-pronator mass. (L, lateral; M, medial; MC, medial capsule.)

The posterolateral “soft spot” portal, located at the center of a triangle formed by the lateral epicondyle, olecranon, and radial head, serves as access for initial joint distention. Approximately 20 mL of saline solution is injected into the joint cavity through the posterolateral “soft spot” to expand the elbow joint. Initially, an incision is made at the planned anterolateral portal located anterior to the radiocapitellar joint. A trocar, along with its sheath, is then inserted through this anterolateral portal while orienting toward both the coronoid process and the medial half of the trochlea (Fig 1). Subsequently, this trocar is replaced with a 4.0-mm 30° arthroscope for inspection. The trochlea, coronoid process, synovium, and the joint capsule are inspected for potential lesions while viewing from the lateral side (Fig 2).

**Fig 3:**
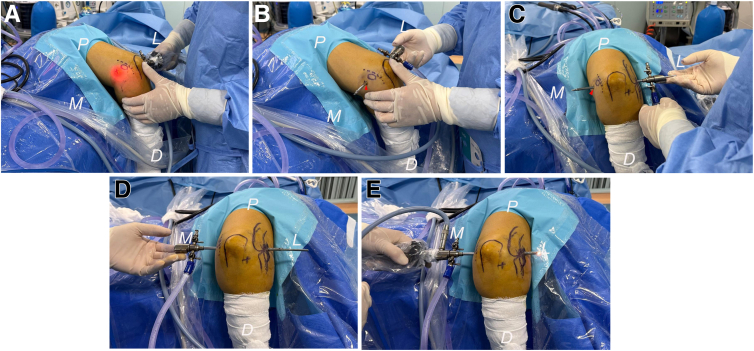
Photographs showing the posterior views of the right elbow with the patient in the lateral position. (A) The anteromedial portal creation follows an inside-out approach. Subcutaneous red illumination becomes visible medially after advancing the arthroscope alongside the trochlea through the medial elbow capsule and flexor muscles. (B) The arthroscope is replaced by a trocar while maintaining its sheath in position, and an incision is made at the anteromedial portal (red arrowhead) using a scalpel over the tip of the trocar; both the trocar and the sheath are subsequently advanced medially out of the skin to create the anteromedial portal. (C, D) The trocar is replaced with a switching stick, facilitating the transfer of both the arthroscope and sheath into the newly created anteromedial portal (red arrowhead). (E) The switching stick trocar is substituted with the arthroscope, which can be retracted into the joint to inspect any pathological conditions on the lateral part of the anterior elbow compartment from a medial perspective via the anteromedial portal. (D, distal; L, lateral; M, medial; P, proximal.)

The anteromedial portal creation follows an inside-out approach; hereafter, the arthroscope is pushed alongside the trochlea and against the medial elbow capsule, as well as further advanced through the capsule and the flexor-pronator mass. Then, the subcutaneous illumination emitted by the arthroscope can be observed medially. Following removal of the arthroscope, it is replaced by a trocar while maintaining its sheath in position, and an incision is made at the anteromedial portal using a scalpel over the skin on the tip of the trocar; both instruments are subsequently advanced medially out of the skin. The trocar is replaced with the switching stick, and the switching stick technique is used for the transfer of the arthroscope along with the sheath into the anteromedial portal (Fig 3).

### Precise Resection of the Lesion Tissue

**Fig 4:**
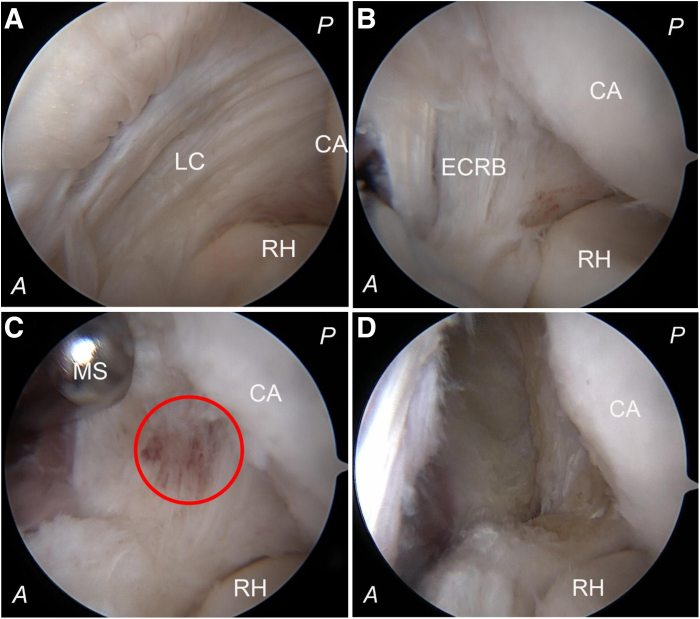
Arthroscopic photographs showing views from the anteromedial portal for the right elbow case. (A) The capitellum, radial head, and the undersurface of the lateral capsule (LC) are first inspected; the lateral capsule is intact, although there seems to be linear capsular tears. According to the Baker lesion classification, it could be classified as type 1 with the intact capsule. (B) The motorized shaver (MS) is introduced into the joint through the anterolateral portal to excise part of the lateral capsule, and the underneath of the ECRB tendon is exposed. (C) Following removal of superficial tendon tissue, a hematoma lesion (red circle) within the ECRB becomes visible. (D) The extensor carpi radialis brevis tendon origin, including the hematoma lesion tissue, is excised, but the equatorial line of the capitellum cannot be observed from the medial view due to blockage by the capitellum, so verification of complete resection cannot be achieved when viewed from the anteromedial portal. (A, anterior; CA, capitellum; ECRB, extensor carpi radialis brevis; P, posterior; RH, radial head.)

The lateral aspect of the anterior elbow compartment, encompassing the capitellum, radial head, and the lateral capsule covering the extensor carpi radialis, is meticulously examined for any pathological conditions while observing from the medial side via the anteromedial portal. A 4.0-mm full radius motorized shaver (Dissector; Arthrex) and a bipolar radiofrequency device (COOLPULSE 90 Electrode; DePuy Mitek) are alternately introduced through the anterolateral portal to excise both the lateral capsule and the extensor carpi radialis brevis tendon. All capsular and lesion tissues located anterior to the equator of the capitellum are thoroughly debrided (Fig 4).

**Fig 5:**
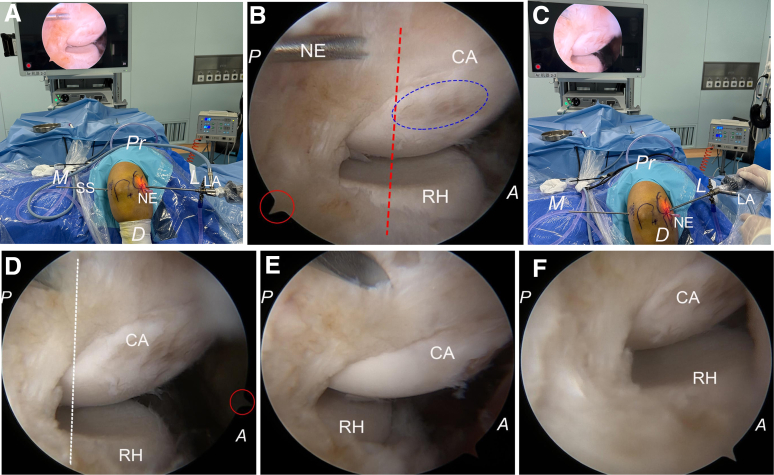
Photographs showing the posterior view of the right elbow with the patient in the lateral position and arthroscopic views of the lateral profile of the capitellum. (A, B) With the elbow at 90° of flexion, the camera is held upright, vertical to the proximal articular surface of the radial head, and the remnant tissue anterior to the equatorial line is marked percutaneously with a needle (NE). The light cable (LA) is turned proximally while the arthroscope is directed distally (red circle). More of the radial head and the capitellum can be visualized, so we get a sideway view, which may result in an anterior misplacement of the equatorial line (red dotted line). Notably, cartilage injury (blue oval circle) on the capitellum during the resection of the lateral capsule is observed. This injury remains obscured when viewed from the medial portal but becomes apparent through observation via the anterolateral portal. (C, D) With the light cable (LA) being turned distally to make the arthroscope orient proximally (red circle), a direct en face view of the lateral profile of the capitellum can be achieved, so the right equatorial line (white dotted line) can be confirmed. (E, F) With elbow extension, visibility extends to the posterior aspect of the capitellum. Conversely, flexion covers its anterior portion. Accurate localization of the equatorial line may be compromised if elbow flexion does not maintain a 90° angle. (A, anterior; CA, capitellum; D, distal; L, lateral; M, medial; Pr, proximal; P, posterior; RH, radial head.)

**Fig 6:**
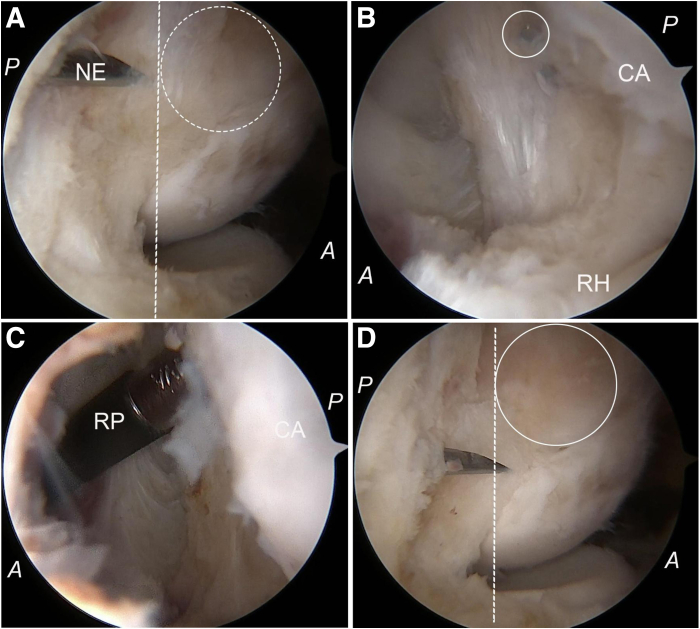
Arthroscopic photographs showing the resection of the remnant tissue on the lateral side of the capitellum. (A) With the arthroscope oriented proximally, the remnant tissue (white dotted circle) on the lateral side of the capitellum, which is still located anterior to the equatorial line (white dotted line), is marked percutaneously using the needle (NE). (B) The arthroscope is transferred back into the anteromedial portal, with the marking needle tip vaguely visible (white circle) by viewing from the medial side. (C) The radiofrequency probe (RP) is inserted through the anterolateral portal again to excise the remnant tissue anterior to the needle tip. (D) To recheck the completeness of resection, the arthroscope is transferred back into the anterolateral portal, and the previously identified remnant tissue (white circle), located anterior to the equatorial line (white dotted line), has been successfully cleared. (A, anterior; CA, capitellum; D, distal; L, lateral; M, medial; P, posterior; RH, radial head.)

To verify the extent of resection achieved, we replace the arthroscope with a switching stick left in situ within the joint; subsequently, we transfer the arthroscope to examine through the anterolateral portal for any residual tissue situated anterior to the equator of the capitellum and distal to the lateral epicondyle. Different portions of the capitellum are exposed during both extension and flexion movements of the elbow, so the elbow is maintained at 90° flexion. The light cable (LA) is turned distally to make the arthroscope orient proximally (red circle), allowing for a direct en face view of the lateral profile of the capitellum to confirm the right equatorial line (Fig 5). Any remaining tissue located anterior to this equatorial line is marked percutaneously using a needle and subjected to further resection while viewing from the medial perspective with reversion of arthroscopic access back to the anteromedial portal (Fig 6). This procedure may be repeated as necessary for precise and complete removal of lesion tissues.

### Postoperative Protocol

Upon completion of the procedure, the portal sites are sutured, and a soft dressing is applied. Elbow range-of-motion exercises are initiated on the first postoperative day with a manageable level of discomfort. Patients may resume daily activities provided that their pain levels remain within acceptable limits; they are generally advised that a return to work can be expected within 6 weeks following the procedure. Pearls and pitfalls of our arthroscopic technique are outlined in Table 1.

**Table 1:** Pearls and Pitfalls

**Pearls**
The creation of the anteromedial portal follows the inside-out technique, with the arthroscope being advanced medially alongside the trochlea to maintain a safe distance from the medial nerve.
To minimize the risk of iatrogenic injury to the medial cutaneous nerve of the forearm, it is crucial that the trocar is pushed against the skin during incision for the anteromedial portal.
The anterolateral portal can be utilized interchangeably as either a working or viewing portal, and visualization through this portal facilitates precise debridement of pre-equatorial capitellar pathology.
Proper proximal orientation of the arthroscope and maintaining 90° flexion of the elbow are essential for accurately locating the equatorial line.
Gentle manipulation of instruments is vital to prevent any iatrogenic damage to cartilage.
**Pitfalls**
Anterior mislocation of the equatorial line of the capitellum with the arthroscope orienting distally.
Inadequate resection performed by less experienced surgeons may elevate recurrence risks due to residual pathological tissue remaining postprocedure.
Excessive resection posteriorly beyond the extensor digitorum communis and lateral collateral ligament.

## Discussion

The advantages of our technique are outlined in Table 2. In this Technical Note, we describe a surgical technique utilizing the anterolateral arthroscopic portal to achieve optimal visualization for assessing ECRB resection extent while enabling concurrent management of intra-articular pathology, reducing persistent postoperative pain, and accelerating recovery. ECRB originates from a diamond-shaped footprint positioned between the articular midline and the superior margin of the humeral capitellum, immediately distal to the supracondylar ridge.^3^^,^^4^

**Table 2:** Advantages and Disadvantages

**Advantages**
This procedure employs a minimally invasive approach.
Arthroscopic visualization facilitates both pathological evaluation and immediate intervention.
The resection of the lesion tissue is clearly visualized and confirmed through the anterolateral portal.
Two portals are adequate for precise lesion tissue resection, which may reduce the incidence of chronic postoperative pain.
**Disadvantages**
This technique necessitates prior expertise in elbow arthroscopy.
A comprehensive 3-dimensional understanding of the surrounding anatomy is paramount for safety and success.

Arthroscopic release of the ECRB tendon has become a well-established treatment for refractory lateral epicondylitis.^5^ Lappen and Siebenlist’s pivotal work^6^ established that arthroscopic ECRB resection achieves functional results equivalent to open procedures while offering the added benefit of simultaneous intra-articular pathology management. Recent technical innovations have further advanced the field. Stiefel and Field’s arthroscopic “bayonet technique” for lateral epicondylitis utilizes a specialized anterolateral portal for direct visualization and sharp ECRB release, showing excellent safety and efficacy in a 198-case series.^7^ In terms of arthroscopic visualization, Gowda et al.^8^ revealed that a 3-portal approach combined with switching stick capsular retraction significantly improves ECRB tendon exposure by establishing a stable surgical corridor and optimizing tissue retraction. Meanwhile, Tsenkov and Dimitrov^9^ proposed an interchangeable portal system that enhances procedural flexibility by allowing seamless switching between proximal anteromedial and anterolateral approaches. However, it is noteworthy that these techniques share a common limitation—the lack of reliable anatomic landmarks for precise intraoperative guidance, which may lead to either excessive resection of healthy tissue or incomplete resection of pathological lesions in clinical practice.

**Fig 7:**
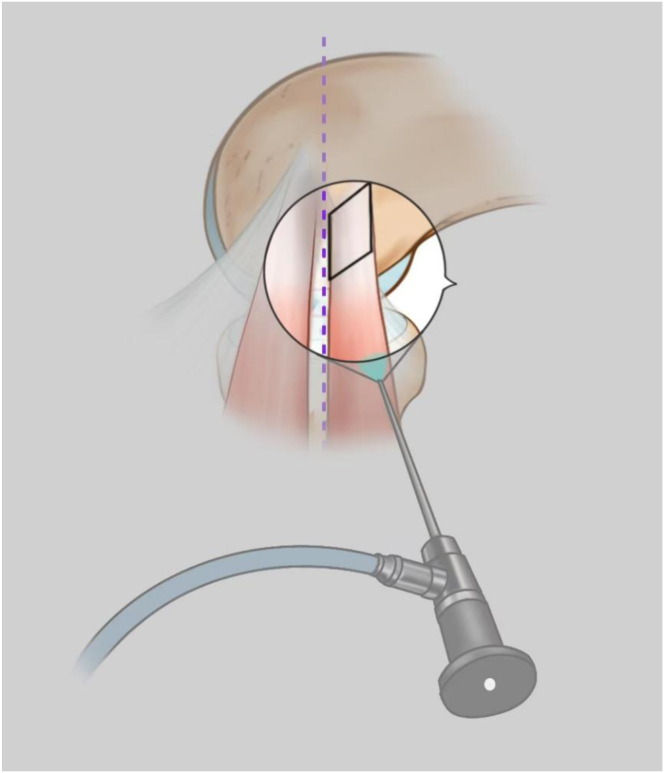
Illustration of arthroscopic view of the extensor carpi radialis brevis with the arthroscope oriented proximally and the equatorial line (purple dotted line) of the capitellum before resection.

We present a surgical technique with direct visualization through the anterolateral portal. By positioning the arthroscope correctly, we can achieve an en face view of the lateral facet of the capitellum, allowing us to verify the equatorial line (Fig 7). Both the anteromedial and anterolateral portals can serve interchangeably as working or viewing portals, enabling precise resection of recalcitrant lateral epicondylitis lesions anterior to the capitellar equator. With proper technique, either excessive resection of healthy tissue or incomplete resection of pathological lesions can be avoided. Precise arthroscopic ECRB resection represents a safe and effective surgical treatment for recalcitrant lateral epicondylitis.

## Disclosures

All authors (C.Z., H-F.G., J-T.M., M-M.D., S-Z.H.) declare that they have no known competing financial interests or personal relationships that could have appeared to influence the work reported in this paper.

## Supplementary Data

Video 1 The lateral position is used for this right elbow case with the elbow at 90° flexion. The oval tender area is outlined prior to anesthesia. The other landmarks and the planned 2 portals are depicted after sterile draping. Saline solution is injected for joint expansion. The anterolateral portal is initially established for inspection of the anterior compartment from the lateral side. Then, the arthroscope is pushed medially alongside the trochlea and passed through the medial capsule and flexor muscles. The subcutaneous illumination emitted by the arthroscope can be observed medially. The arthroscope is then replaced with a trocar, and the anteromedial portal is made using a scalpel over the tip of the trocar. The switching stick technique is used for the transfer of the arthroscope into the anteromedial portal. The lateral aspect of the anterior compartment is inspected while viewing from the medial side. The shaver and the radiofrequency device are alternately introduced via the anterolateral portal to remove the lateral capsule and the extensor carpi radialis brevis tendon, and the hematoma lesion in the extensor carpi radialis brevis tendon is exposed and removed. To verify completeness of the resection, the arthroscope is transferred to the anterolateral portal to assess any residual tissue remaining anterior to the equator of the capitellum. The remnant tissue is marked percutaneously with a needle. With elbow extension and flexion, the exposure of the capitellum varies, so elbow flexion at 90° is very important for locating the equatorial line. The camera is held upright, and the arthroscope is turned distally. More of the radial head and the capitellum can be visualized, so we get a sideway view, which may result in an anterior misplacement of the equatorial line. With the arthroscope oriented proximally, a direct en face view of the lateral profile of the capitellum can be achieved, so the right equatorial line can be confirmed. The remnant tissue anterior to the marking needle and the equator is resected again by viewing from the medial side with the arthroscope transferred back to the anteromedial portal. With the switching stick technique being used again, the arthroscope is transferred back to the anterolateral portal to recheck the completeness of the resection. This procedure can be repeated for precise and thorough resection of the lesion tissue.
